# In Vitro Animal Model for Estimating the Time since Death with Attention to Early Postmortem Stage

**DOI:** 10.3390/metabo13010026

**Published:** 2022-12-23

**Authors:** Michal Szeremeta, Paulina Samczuk, Karolina Pietrowska, Tomasz Kowalczyk, Katarzyna Przeslaw, Julia Sieminska, Adam Kretowski, Anna Niemcunowicz-Janica, Michal Ciborowski

**Affiliations:** 1 Department of Forensic Medicine, Medical University of Bialystok, 15-269 Bialystok, Poland; 2 Metabolomics Laboratory, Clinical Research Center, Medical University of Bialystok, 15-276 Bialystok, Poland; 3 Department of Physical Chemistry, Medical University of Bialystok, 15-328 Bialystok, Poland; 4 Department of Endocrinology, Diabetology and Internal Medicine, Medical University of Bialystok, 15-276 Bialystok, Poland

**Keywords:** postmortem interval, plasma, metabolomics, LC-MS, porcine animal model

## Abstract

Estimating the postmortem interval (PMI) has remained the subject of investigations in forensic medicine for many years. Every kind of death results in changes in metabolites in body tissues and fluids due to lack of oxygen, altered circulation, enzymatic reactions, cellular degradation, and cessation of anabolic production of metabolites. Metabolic changes may provide markers determining the time since death, which is challenging in current analytical and observation-based methods. The study includes metabolomics analysis of blood with the use of an animal model to determine the biochemical changes following death. LC-MS is used to fingerprint postmortem porcine blood. Metabolites, significantly changing in blood after death, are selected and identified using univariate statistics. Fifty-one significant metabolites are found to help estimate the time since death in the early postmortem stage. Hypoxanthine, lactic acid, histidine, and lysophosphatidic acids are found as the most promising markers in estimating an early postmortem stage. Selected lysophosphatidylcholines are also found as significantly increased in blood with postmortal time, but their practical utility as PMI indicators can be limited due to a relatively low increasing rate. The findings demonstrate the great potential of LC-MS-based metabolomics in determining the PMI due to sudden death and provide an experimental basis for applying this attitude in investigating various mechanisms of death. As we assume, our study is also one of the first in which the porcine animal model is used to establish PMI metabolomics biomarkers.

## 1. Introduction

A forensic investigation’s daily task is to estimate the time since death (postmortem interval—PMI). While performing external examinations of corpses, PMI is determined mainly by evaluating early postmortem changes (livor mortis, rigor mortis, and algor mortis) and supravital reactions. The early postmortem phase is claimed to be the most crucial period for PMI estimation, while most medico-legal cases are examined. The purpose of assessing the time of death is to help establish the objective truth, which in criminal proceedings relates to verifying witnesses’ testimonies and confirming or undermining the suspect’s alibi. This period is also crucial when the estimation of PMI is most relevant in cases such as establishing the timeline of events and developing the theory of death circumstances.

As forensic medical practice shows, conclusions of the postmortem assessment of the time since death can be very diverse and depend on many internal (diseases that the individual suffered during their lifetime) and external (temperature and humidity) factors affecting the body. Therefore, to this day, researchers have been using several experimental methods to estimate PMI precisely, which still do not show the expected results.

Due to the medico-legal limitations related to the assessment of the classical triad of postmortem changes (rigor mortis, livor mortis, and algor mortis), methods from various fields, such as chemistry and biochemistry, histopathology and immunohistochemistry, molecular biology, and entomology have been used to assess the PMI [1,2,3,4,5,6,7,8,9]. At the same time, tissues and organs, mainly the heart, liver, kidney, blood, and other body fluids have been the subject of research. Visible examples are cerebrospinal fluid, synovial fluid, and the eye’s vitreous humor [10,11,12,13,14,15]. The latest methods used to estimate PMI include microbiome analysis, i.e., of the human microbiota [16,17], or the newest omics technologies such as metabolomics [18]. The studies concerning PMI estimation by the metabolomics approach have a relatively modest medico-legal history [18]. To date, these have included assessing metabolite changes in the animal brain, heart, liver, spleen, and dorsal muscle [19,20]. Additionally, postmortem changes in metabolites in animal and human blood have been studied [21,22]. The latest studies in this area are based on animal models, which means that metabolomics techniques in determining the PMI still require testing under standardized conditions [23,24].

In the present study, one of the metabolomics techniques, liquid chromatography combined with mass spectrometry (LC-MS), was used to indicate novel plasma biomarkers, allowing the estimation of the time since death in the early postmortem stage, in a porcine model. Blood samples were collected immediately after death with anticoagulant (EDTA) and without (non-EDTA samples). Such an experimental design allowed us to simulate sudden and not sudden death situations. In case of sudden death, blood remains fluid, caused by a high fibrinolytic activity due to the release of plasminogen activator from the vascular wall [25]. In this way, we can check if postmortal metabolic changes in the blood are affected by blood fluidity, and we can search the universal candidates for metabolic biomarkers in an early postmortem stage.

## 2. Material and Methods

### 2.1. Animals and Main Experiment Criteria

The experiment was performed on animal blood samples (domestic pigs, breed: Polish Large White). Pigs were kept under traditional free-range rearing and ate a mixed diet consisting of soybean and corn. The mature pigs (weight range 300–400 kg) died of hemorrhagic shock. The blood from the animals was secured at the time of slaughter. The incision, resulting in fatal bleeding, was made in the neck area. In total, sixteen animals took part in the study, and blood samples from eight animals were collected with EDTA, while blood samples from another eight were collected without EDTA. Blood was routinely obtained from the carotid artery and collected into two special containers intended for this purpose (one with EDTA and the other without it). Within an hour since the animal died, blood was delivered to the isolated room in the Department of Forensic Medicine Division, Medical University of Bialystok, and split equally into sterile cups. Immediately after the blood was split, samples from the first cups were collected and marked as the starting point (time 0 h). The experiment was conducted for 7 days, but in this study, the data for the following time points are presented: 0 h, 3 h, 6 h, 9 h, 12 h, 18 h, and 24 h (in total, 112 blood samples were included in this study). At each time point, blood samples with anticoagulant (K_2_EDTA), described as EDTA samples, were collected in 10 mL tubes, while blood samples without anticoagulant, described as non-EDTA samples, were collected in 9 mL vacuum system tubes with K_2_EDTA. Both tubes were gently mixed and immediately centrifuged at 1900× *g* for 10 min at 4 °C. Then, plasma fractions were collected in Eppendorf tubes and centrifuged again (10,000× *g* for 15 min at 20 °C). Finally, plasma samples were collected in new Eppendorf tubes and stored at −80 °C until the analysis day. The experiment was conducted in stable conditions with constantly monitored temperature and humidity. One research team member entered the room only to collect the samples at selected time points.

### 2.2. Ethics

Experiments performed on the blood of slaughtered animals do not require the Local Ethics Committee’s consent, which was confirmed by the decision (LKE 20/2021) of the Local Ethical Committee for Animal Experiments in Olsztyn (Poland).

### 2.3. Procedures

#### 2.3.1. Chemicals and Reagents

Purified water was obtained by using a Milli-Q Integral 3 system (Millipore SAS, Molsheim, France). Zomepirac sodium salt (used as internal standard, IS), L-pyroglutamic acid, hypoxanthine, L-histidine monohydrochloride monohydrate, oleic acid, L(+) lactic acid, LS-MS grade acetonitrile, methanol, formic acid, and LC grade ethanol were purchased from Sigma-Aldrich Chemie GmbH (Steinheim, Germany). The API-TOF reference mass solution kit (G1969-850001) and tuning solutions, ESI-L low-concentration tuning mix (G1969-85000), and ESI-TOF Biopolymer Analysis reference masses (G1969-850003) were purchased from Agilent Technologies (Santa Clara, CA, USA).

#### 2.3.2. Sample Processing

Plasma samples were treated as described previously [26]. Briefly, protein precipitation and metabolite extraction were performed by vortex-mixing one volume of the plasma sample for 1 min with four volumes of an ice cold (−20 °C) methanol/ethanol (1:1) mixture containing 1 ppm of zomepirac. After extraction, samples were stored on ice for 10 min, centrifuged at 21,000× *g* for 20 min at 4 °C, and the supernatant was filtered through a 0.22 µm nylon filter into glass vials. Quality control (QC) samples were prepared by mixing an equal volume of all samples. The obtained mixture was prepared following the same procedure as the other samples.

### 2.4. Assays

#### 2.4.1. Plasma Metabolic Fingerprinting

Metabolic fingerprinting was performed by a 6550 iFunnel LC-Q-TOF-MS (Agilent Technologies, Santa Clara, CA, USA) system. Plasma samples were analyzed according to the previously described protocols [26,27]. Samples were randomly analyzed by an LC-MS system consisting of 1290 Infinity LC with a degasser, two binary pumps, and a thermostatted autosampler combined with a 6550 iFunnel Q-TOF-MS detector (both Agilent Technologies, Santa Clara, California, USA). Analyses were performed in positive (ESI+), and negative (ESI-) ion modes, whereby 1μL of the sample was injected into a thermostatted (60 °C) Zorbax Extend-C18 RRHT (2.1 × 50 mm, 1.8 μm particle size, Agilent Technologies) chromatographic column. The flow rate was 0.6 mL/min with solvent A (water with 0.1% formic acid) and solvent B (acetonitrile with 0.1% formic acid). The chromatographic gradient started at 5% of phase B for the first minute, followed by an increase in phase B to 80% (from 1 to 7 min) and to 100% (from 7 to 11.5 min). The system was re-equilibrated by reverting phase composition to initial conditions (5% phase B) in 0.5 min, which was kept from 12 to 15 min. The mass spectrometer was operated in full scan mode from mass m/z 50–1000. The capillary voltage was set to 3 kV for positive and 4 kV for negative ionization mode; nozzle voltage was 1000 V; the drying gas flow rate was 12 L/min at 250 °C and gas nebulizer at 52 psig; the fragmentor voltage was 250 V for positive and negative ionization modes. Data were collected in centroid mode at a scan rate of 1.5 scans per second. Accurate mass measurements were obtained using calibrant solution delivery using a dual-nebulizer ESI source. A calibrating solution containing reference masses at m/z 121.0509 (protonated purine) and m/z 922.0098 (protonated hexakis (1H,1H,3H-tetrafluoropropoxy)phosphazine or HP-921) in positive ion mode or m/z 119.0363 (proton abstracted purine) and m/z 966.0007 (formate adduct of HP-921) in negative ion mode was continuously introduced by an isocratic pump (Agilent, Santa Clara, CA, USA) at a flow rate of 0.5 mL/min (1:100 split).

#### 2.4.2. LC-MS Data Processing

The raw data collected by the analytical instrumentation were cleaned of background noise and unrelated ions by the molecular feature extraction (MFE) tool in Mass Hunter Qualitative Analysis Software (B.07.00, Agilent, Santa Clara, CA, USA). The MFE creates a list of all possible components described by mass, retention time (RT), and abundance. The background noise threshold was set to 400 counts. The following adduct settings were applied: +H, +Na, +K in positive ion mode, and −H, +HCOO, +Cl for negative ion mode. Both were used to identify co-eluting adducts of the same feature. Dehydration neutral losses were also allowed in both ionization modes. Sample alignment and data filtering were performed using Mass Profiler Professional 12.6.1 (Agilent, Santa Clara, CA, USA). Parameters applied for the alignment were 1% for RT and 20 ppm for the mass variation. In the quality assurance (QA) procedure, metabolic features detected in >50% of QC samples with the coefficient of variation (CV) <25% were kept. Subsequently, metabolites present in at least 80% of the samples from at least one of the compared groups were analyzed statistically. Additionally, missing values were replaced as described by Armitage et al. [27].

#### 2.4.3. Statistics

To select statistically significant metabolic features, two-group comparisons (0 vs. 3, 0 vs. 6, 0 vs. 9, 0 vs. 12, 0 vs. 18, 0 vs. 24, for EDTA and non-EDTA separately) were performed using univariate statistics. The Shapiro–Wilk test was used for normality testing, and then, depending upon data distribution, a *t*-test or Mann–Whitney U test was performed. Obtained *p*-values were corrected by Benjamini–Hochberg false discovery rate (FDR). The level of statistical significance was set at 95% (*p* < 0.05). Univariate statistical analyses were performed in MATLAB (R2015a). Multivariate calculations and plots were performed by using SIMCA−P + 13.0.3.0 (Umetrics, Umeå, Sweden) to evaluate data quality by checking the QC samples’ location on principal component analysis (PCA) plots and observing sample discrimination on partial least squares discriminant analysis (PLS-DA) plots.

#### 2.4.4. Metabolite Identification

Identification of metabolites was performed based on the MS/MS fragmentation as previously described [28,29]. Accurate masses of features were searched against the METLIN, KEGG, LIPIDMAPS, and HMDB databases, which were simultaneously accessed by CEU Mass Mediator (available online, http://ceumass.eps.uspceu.es/mediator/ (accessed on 17 March 2021). The identity of metabolites was confirmed by matching the experimental MS/MS spectra to MS/MS spectra from databases or with the fragmentation spectra and retention time obtained for the metabolite’s standard. The identity of the following metabolites was confirmed by the analysis of the standards: pyroglutamic acid, hypoxanthine, histidine, oleic acid, and lactic acid. Experiments were repeated with identical chromatographic conditions to the primary analysis. Ions were targeted for collision-induced dissociation (CID) fragmentation on the fly based on the previously determined accurate mass and retention time. Phospholipids and acylcarnitines with typic fragmentation patterns were identified based on previously described characteristic fragments [30,31].

#### 2.4.5. Metabolic Pathways Analysis

The pathways analysis was performed with MetaboAnalyst 5.0 (http://www.metaboanalyst.ca/ (accessed on 23 March 2021). This online tool analyzes particular compounds’ impact on biochemical pathways, specifically for metabolomics studies [32].

## 3. Results and Discussion

During the experiment, stable environmental conditions were maintained, with temperature and humidity varying from 21.5–22.0 °C and 36.98–40.42%, respectively.

**Figure 1:**
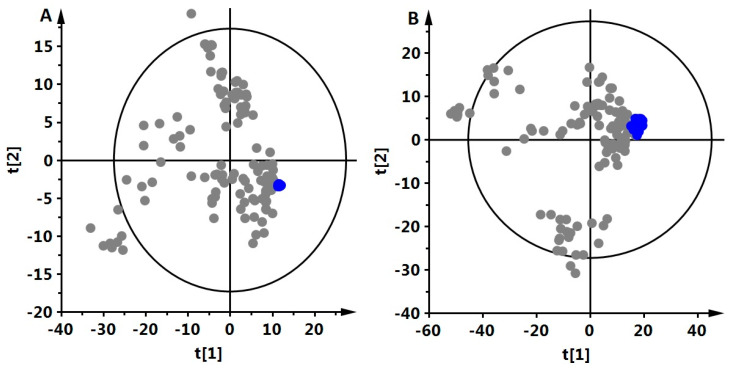
Classification of QC samples (marked in blue) in a PCA model.

**Figure 2:**
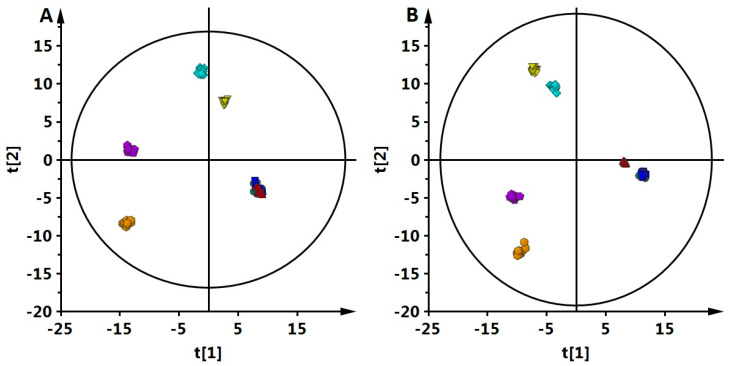
A PLS-DA score plot was obtained for all time points.

Plasma samples from 16 (eight each of EDTA and non-EDTA) animals and 7 time points (from 0 h to 24 h) were fingerprinted with LC-MS. As the experiment was performed with EDTA and non-EDTA samples, 112 plasma samples were analyzed. EDTA and non-EDTA samples were analyzed together, first in positive ion mode and later in a negative one. Consequently, two data sets were obtained. The QA procedure was performed for obtained data sets, resulting in 469 metabolic features in positive and 670 in negative ionization mode, respectively. Using those data, PCA was performed to check the classification of the QC samples. Close clustering of QCs in obtained PCA models (Figure 1) indicates the system’s stability and performance during analyses. To illustrate the studied samples’ classification based on obtained plasma fingerprints, PLS-DA plots for all time points (samples EDTA and non-EDTA together) were made (Figure 2). Statistical analysis was performed to indicate significant metabolic features, giving 181 (ESI+) and 122 (ESI-) metabolic features significant in at least one of the comparisons. Finally, 51 metabolites were identified. Significant and identified metabolites from all performed comparisons are gathered in supplementary tables. Table S1 contains information about the level of change for each metabolite in each comparison and respective *p*-value, while Table S2 contains monoisotopic mass, retention time, calculated error of measured mass, and MS/MS fragments for identified metabolites.

Panel A shows a PCA model based on data generated in positive ion mode while panel B is in negative ion mode. Data were centroid-scaled and log-transformed.

Panel A shows data for positive ion mode (R2 = 0.887, Q2 = 0.584) while panel B shows negative ion mode (R2 = 0.832, Q2 = 0.826). All data were Pareto-scaled and log-transformed. Green circle—0 h, dark blue box—3 h, red triangle—6 h, yellow inverted triangle—9 h, blue diamond—12 h, violet pentagon—18 h, and orange hexagon—24 h. Presented results include data from EDTA and non-EDTA samples together.

**Figure 3:**
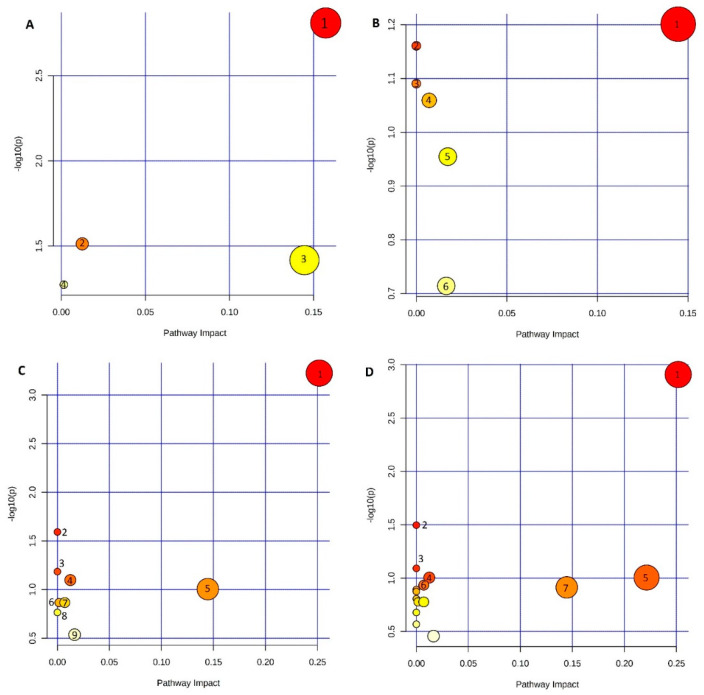
Metabolic pathway analysis performed for metabolites significant in different comparisons: 0 vs. 6 (panel **A**), 0 vs. 9 (panel **B**), 0 vs. 12 (panel **C**), and 0 vs. 18 (panel **D**). Pathway analysis includes data from comparisons with EDTA and non-EDTA together. Significant pathways marked with the numbers are panel A—1. Glycerophospholipid metabolism, 2. Glycerolipid metabolism, 3. Ether lipid metabolism, and 4. Phosphatidylinositol signaling system; panel B—1. Ether lipid metabolism, 2. Pyruvate metabolism, 3. Glycolysis/Gluconeogenesis, 4. Glutathione metabolism, 5. Glycerophospholipid metabolism, and 6. Purine metabolism; panel C—1. Glycerophospholipid metabolism, 2. Linoleic acid metabolism, 3. alpha-Linolenic acid metabolism, 4. Glycerolipid metabolism, 5. Ether lipid metabolism, 6. Phosphatidylinositol signaling system, 7. Glutathione metabolism, 8. Biosynthesis of unsaturated fatty acids, and 9. Purine metabolism; panel D—1. Glycerophospholipid metabolism, 2. Linoleic acid metabolism, 3. alpha-Linolenic acid metabolism, 4. Glycerolipid metabolism, 5. Histidine metabolism, 6. Pantothenate and CoA biosynthesis, and 7. Ether lipid metabolism.

A pathway analysis for each comparison was performed using MetaboAnalyst 5.0 (Figure 3) to give an overview of the metabolic pathways to which identified metabolites belong. There were no significant metabolites for 0 vs. 3 comparisons, while for 0 vs. 18 comparisons, the obtained result was comparable to 0 vs. 24 comparisons (data not shown). Glycerophospholipid metabolism, as well as other lipid metabolism pathways, was standard in every comparison.

The most desirable candidates for biomarkers for the PMI estimation, including the early postmortem stage, would be the compounds in which postmortem concentrations in blood change significantly with minimal data scattering, relatively slowly and monotonously (or, even better, linearly). Our study showed 51 significant metabolites that can help estimate the time since death in the early postmortem stage. Many of them are associated with glycerophospholipid metabolism and metabolism of other lipids. The presence of metabolites associated with lipid metabolism, including glycerophospholipid metabolism, is primarily related to cell membrane changes [33]. Moreover, after death, the fastest changes correspond to the disruption of most energy-dependent metabolic processes as an answer to the lack of oxygen. The cell anoxia can also cause an increase in the intracellular osmotic pressure due to the dysfunction of Na^+^/K^+^ pumps, cell lysis with membrane degradation, and cytoplasm leakage into intercellular space [34,35].

**Figure 4:**
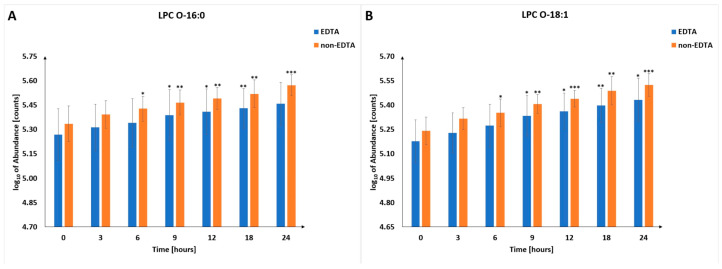
Changes in the level of selected lysophosphatidylcholines in different postmortem time intervals. Panel (**A**) shows the trajectory for LPC O-16:0, while panel (**B**) shows LPC O-18:1. *—*p*-value < 0.05, **—*p*-value < 0.01, ***—*p*-value < 0.001.

**Figure 5:**
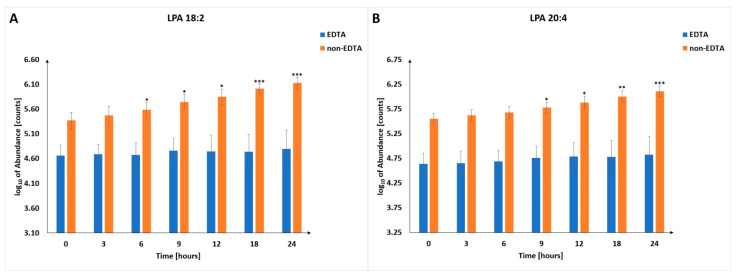
Changes in the level of selected lysophosphatidic acids in different postmortem time intervals. Panel (**A**) shows the trajectory for LPA 18:2, while panel (**B**) shows LPA 20:4. *—*p*-value < 0.05, **—*p*-value < 0.01, ***—*p*-value < 0.001.

Due to cell lysis and membrane degradation, changes in the level of phospholipids are observed (Table S1). The majority of significantly changing phospholipids are lysophosphatidylcholines (LPCs), with some (Figure 4) increasing similarly in EDTA and non-EDTA samples. These metabolites can be considered as candidates for universal metabolic markers of PMI. Among others, lysophosphatidic acid (LPA) can be mentioned. Once thought to be an inert metabolite in the biosynthesis of membrane phospholipids, LPA is now well recognized as an essential signaling molecule. There are two major pathways of LPA production. The first one involves the hydrolysis of phosphatidic acids (PAs) by phospholipases A1 and A2 (PLA1 and PLA2) [36]. The second pathway leads through cleavage of lysophospholipids (LPLs), such as LPC and lysophosphatidylserine (LPS), by lysophospholipase D/autotaxin (LysoPLD/ATX) [37]. However, LPA produced by the above-described pathways appears to serve as a precursor for glycerolipid synthesis rather than a source of extracellular signaling molecules [38]. On the other side, elevated LPA levels are visible during ischemia and hypoxia, which are significant causes of damage to human cells. During ischemia and hypoxia, LPA appears to play a protective role and has been shown to protect different cell types from hypoxia-induced apoptosis, including cardiac myocytes, mesenchymal stem cells, and renal cells [39,40,41]. We have observed changes in two LPAs (18:2 and 20:4) which significantly increased in the samples collected without adding EDTA (Figure 5).

It is well known that postmortem oxygen deficiency results in pyruvate’s fast decay. A significant elevation of fumarate, succinate, and hypoxanthine levels is also a consequence. The concentrations of these compounds in blood undergo significant changes just after death. Postmortem anaerobic glycolysis leads to lactate accumulation and glucose consumption. The accumulation of acidic metabolites in the blood is also the result of the following postmortem systemic imbalance—a change in blood pH depends on acid–base buffers such as carbonic acid and bicarbonate ion applying their influence principally through the kidneys and the respiratory system to control the acid–base balance [42]. In deceased persons, the blood pH changes occur because the body’s buffering system is not maintained [43].

Another group of metabolites showing a postmortem increase because of protein degradation are amino acids. The twenty natural amino acids are the building blocks of three-dimensional protein structures. Each has unique structural characteristics and physicochemical properties and plays an irreplaceable role in proteins’ biochemistry and biological functions. Histidine may be the most versatile actor in protein architectures and bioactivities among natural amino acids [44,45,46].

**Figure 6:**
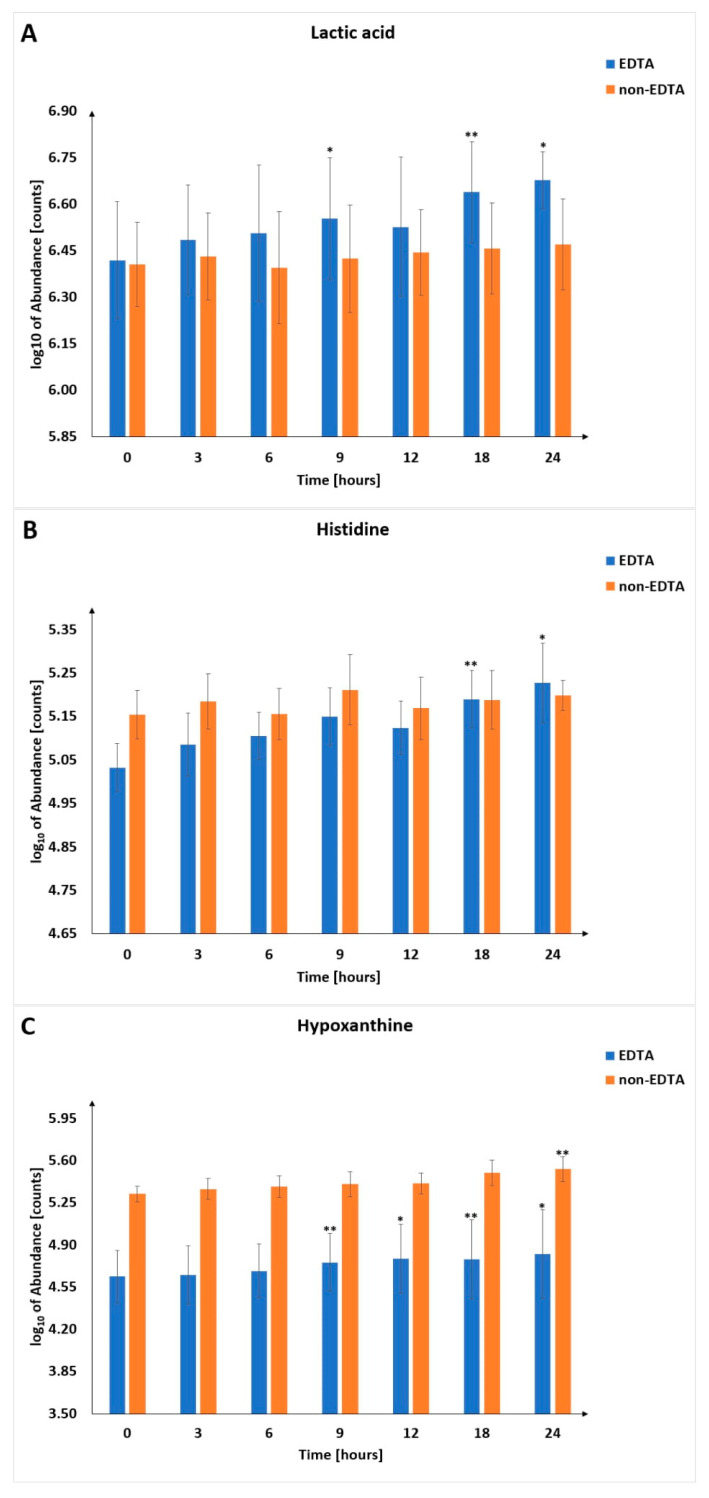
Changes in the level of selected metabolites in different postmortem time intervals. Panel (**A**) shows the trajectory for lactic acid, panel (**B**) shows histidine, and panel (**C**) shows hypoxanthine. *—*p*-value < 0.05, **—*p*-value < 0.01.

Due to the above-described processes and individual natures of the compounds, we have indicated the usefulness of selected metabolites (hypoxanthine, lactic acid, histidine, and lysophosphatidic acids), which, based on obtained results, are the most promising metabolic indicators of PMI. Lactic acid, histidine, and hypoxanthine (Figure 6) were found to be better indicators of PMI in samples with EDTA, while LPAs were better in samples without EDTA. Selected LPCs were found consistently and significantly increased in both types of samples (Figure 4). Nevertheless, the magnitude of change was much lower than in the case of the metabolites mentioned above (Figure 5 and Figure 6), which may affect their practical utility as PMI indicators.

### 3.1. Hypoxanthine

Hypoxanthine (Hx), a vital degradation product of adenosine nucleotide metabolism, is formed by several enzymatic reactions, especially during terminal stages of purine catabolism in humans. Hx concentration in the blood increases after death because of hypoxia, promoting Hx generation from adenosine monophosphate (AMP) [47,48]. Hypoxanthine has already been considered a potential marker of hypoxia and PMI for many years, especially in anatomically isolated fluids, such as ocular fluids: aqueous humor (AH) and vitreous humor (VH) [15,49,50,51]. AH and VH have advantages over blood PMI biomarkers because postmortem metabolomic changes are affected by many processes in the body. Despite this, a positive linear correlation of serum Hx concentration according to PMI with Pearson’s coefficient r > 0.5 has been recently found by Zelentsova et al. [52]. In this research, hypoxanthine level in blood increases from approximately 25–60 nmol/g at 10 min postmortem to 350–550 nmol/g at 4 h postmortem. In earlier data, Zelestova et al. showed that Hx level increased 8-fold in serum 10 min after death [34]. Changes observed for hypoxanthine most likely correspond to the early postmortem events related to breaking the tricarboxylic acid (TCA) cycle and purine catabolism in an oxygen-deficient biological medium. Elevated hypoxanthine concentrations are also found in plasma by Boulieu et al. and Poulses et al. [53,54]. There is evidence that Hx concentration increases linearly for the first 24 h after death in humans and animals [55,56]. According to PMI, a constant increase in serum Hx level is also found in our data (significant at several time points for blood samples with an anticoagulant), which means that hypoxanthine can be a better PMI estimator when the blood remains fluid (Figure 6C).

### 3.2. Lactic Acid

The concentration of lactic acid (lactate) increases after death under anaerobic conditions, considering the absence of an oxygen supply. During carbohydrate metabolism under anaerobic conditions, pyruvate is metabolized to lactic acid by glycolysis [57]. The lactate production is not only glycolysis but also autolysis and bacterial catabolism [58]. Circulating serum lactate in human heart blood was studied postmortem and increased in the blood 20-fold one hour after death and then rose 50–70 times higher than antemortem levels by 24 h [59]. The lactate concentrations in the postmortem blood may be affected by lactate diffusion from muscular tissues and glycolysis in the vessels. That makes lactic acid an unsatisfactory marker for PMI [60]. More recent studies disagree with this opinion because lactate concentration increased significantly (*p*-value < 0.0001) by 40-fold postmortem [22]. In the study by Donaldson and Lamont on in vivo animal model, lactate concentration rose rapidly from 0.5 to 2.5 mmo/L immediately postmortem to between 3 and 7.5 mmol/L by three hours postmortem. The lactate concentration hit a plateau by 9 h [21]. The increase in lactate concentrations in the plasma following death most likely reflects cellular lysis from tissues such as red blood cells and skeletal muscles. Lactate accumulates in the bloodstream as it is not synthesized into glucose via gluconeogenesis or converted to pyruvate by lactate dehydrogenase due to the absence of the NAD^+^ co-factor. A moderate growth during the first hour postmortem is demonstrated by the lactate levels in serum. Sato et al. found that lactic acid levels increased at the 6 h point [61]. A similar observation was visible during a previous study by Katsumata et al. [62]. The usefulness of lactate in estimating PMI has been reported in our studies, especially for blood samples with an anticoagulant (Figure 6A).

### 3.3. Histidine

The total protein concentration increases time-dependently but not significantly and remains within the range of antemortem values [63,64]. The situation is different with amino acids (the protein components). The postmortem increase in free amino acid concentration may be due to several possible and complementary causes. The first is associated with postmortem protein catabolism and cell lysis. The latter shows that each tissue contains different concentrations of free intracellular amino acids and releases different amounts of each amino acid into the blood through cellular lysis [22]. The third possibility is decreased protein synthesis, which is part of hypoxia’s metabolic adaptation. ATP-consuming reactions, such as protein synthesis, are dramatically decreased before ceasing altogether [65]. This decrease in protein synthesis means that the free amino acids are used in protein synthesis to accumulate in the bloodstream as cells lyse. Cells no longer synthesize ATP during death, so amino acids collect intracellularly and are released into the bloodstream through cellular lysis. A statistically significant increase in the amino acid level is observed in the animal model prepared by Jawor et al. [33]. A higher level of amino acids may originate from hypoxia [66], postmortem protein degradation [67] due to cell lysis, and ongoing anabolic–catabolic processes that are strongly imbalanced postmortem. The monotonous growth of free amino acids with the PMI increase was described in previous experiments [22,34,52]. For example, Donaldson and Lamont stated that six amino acids have relative concentration changes of 50–500-fold postmortem, and nine amino acids (including histidine) have relative concentration changes of 46–128-fold postmortem [22]. Zelestova et al. showed that histidine is a metabolite with weak time correlation and moderate data scatter [52]. The usefulness of histidine in estimating PMI has also been reported in our study, especially for blood samples with an anticoagulant (Figure 6B).

### 3.4. Lysophosphatidic Acids (LPAs)

LPA is present in all fluids and eukaryotic tissues under physiological and pathophysiological conditions [68,69,70,71,72,73]. LPA is the smallest bioactive lipid that exerts potent extracellular signaling through its interaction with its six specific G protein-coupled receptors (GPCRs), mediating critical responses such as cell proliferation, migration, and cytoskeletal reorganization [74,75]. LPA can be present both intra- and extracellularly. There is one major pathway involved in LPA extracellular synthesis. Membrane phospholipids, such as phosphatidylcholine, phosphatidylserine, and phosphatidylethanolamine, are the precursor molecules to produce lysophosphatidylcholine (LPC), lysophosphatidylethanolamine, and lysophosphatidylserine (LPS), respectively, through the action of phospholipase A1 (PLA1) or PLA2. These lysophospholipids (LPs) are then converted into LPA by autotoxin (ATX), which is mainly responsible for LPA maintenance at a physiological concentration in plasma [76]. Regarding intracellular LPA production, at least four pathways are involved in this process: the monoacylglycerol kinase (MAGK) pathway [37,77]; the phosphatidic acid–phospholipase A1 (PA-PLA1) or A2 (PA-PLA2) pathway [36]; the glycerophosphate acyltransferase (GPAT) synthesis pathway [78]; the oxidative modification of low-density lipoprotein (LDL) pathway [79,80]. Additionally, LPA is intermediate in glycerolipid synthesis, produced by GPAT-mediated conversion of glycerol-3-phosphate (G3P) [38]. Finally, LPA production’s intracellular mechanisms can also be essential for its degradation: generation of MAG by LPPs; conversion into PA by AGPTA; G3P production by lysophosphatases/lysophospholipases [37,77]. It is also important to note that most current evidence suggests that intracellular LPA synthesis and degradation serve as a precursor source for glycerolipid synthesis and not extracellular signaling molecules [81].

To the best of our knowledge, these are the first data that indicate that LPAs (18:2 and 20:4) can be used as PMI indicators. The disturbances in the synthesis and degradation of LPA, associated with the dying process and the postmortem interval, may be consistent enough to determine the time of death. The development of research in this area should help understand the roles of LPA in PMI estimation.

## 4. Conclusions

All aspects considered, we prove the usefulness of LC-MS-based metabolic fingerprinting in estimating the time since death. The most promising PMI markers in the early postmortem stage are hypoxanthine, lactic acid, histidine, and lysophosphatidic acids. LPAs are found to be better PMI indicators in the case of non-EDTA samples, while others in the case of EDTA samples. Selected LPCs are found as universal PMI indicators, but a relatively low increasing rate can limit their practical utility. As our examination was performed on an animal model, further studies are obligatory to validate the use of these potential biomarkers in humans. Due to the potential of the obtained results, metabolomics should be considered a valuable tool to estimate postmortem interval.

## Data Availability

The raw data supporting the conclusion of this article will be available upon request in justified cases.

## Supplementary Materials

The following supporting information can be downloaded at: https://www.mdpi.com/article/10.3390/metabo13010026/s1, Table S1: The list of statistically significant metabolites; Table S2: The details of the identification of statistically significant metabolites.
